# Identification of common and cell type specific LXXLL motif EcR cofactors using a bioinformatics refined candidate RNAi screen in *Drosophila melanogaster *cell lines

**DOI:** 10.1186/1471-213X-11-66

**Published:** 2011-11-03

**Authors:** Melissa B Davis, Inigo SanGil, Grace Berry, Rashidat Olayokun, Lori H Neves

**Affiliations:** 1 Department of Genetics, University of Georgia, Athens GA, 30502, USA; 2 Department of Biology, University of New Mexico, Albuquerque, NM 87131, USA; 3 Columbia University School of Medicine, New York, New York, USA; 4 School of Public Health, Southern Connecticut University, New Haven, CT 06511, USA; 5 Department of Human Genetics, Yale University School of Medicine New Haven, CT 06511, USA

## Abstract

**Background:**

During *Drosophila *development, titers of the steroid ecdysone trigger and maintain temporal and tissue specific biological transitions. Decades of evidence reveal that the ecdysone response is both unique to specific tissues and distinct among developmental timepoints. To achieve this diversity in response, the several isoforms of the Ecdysone Receptor, which transduce the hormone signal to the genome level, are believed to interact with tissue specific cofactors. To date, little is known about the identity of these cofactor interactions; therefore, we conducted a bioinformatics informed, RNAi luciferase reporter screen against a subset of putative candidate cofactors identified through an *in silico *proteome screen. Candidates were chosen based on criteria obtained from bioinformatic consensus of known nuclear receptor cofactors and homologs, including amino acid sequence motif content and context.

**Results:**

The bioinformatics pre-screen of the *Drosophila melanogaster *proteome was successful in identifying an enriched putative candidate gene cohort. Over 80% of the genes tested yielded a positive hit in our reporter screen. We have identified both cell type specific and common cofactors which appear to be necessary for proper ecdysone induced gene regulation. We have determined that certain cofactors act as co-repressors to reduce target gene expression, while others act as co-activators to increase target gene expression. Interestingly, we find that a few of the cofactors shared among cell types have a reversible roles to function as co-repressors in certain cell types while in other cell types they serve as co-activators. Lastly, these proteins are highly conserved, with higher order organism homologs also harboring the LXXLL steroid receptor interaction domains, suggesting a highly conserved mode of steroid cell target specificity.

**Conclusions:**

In conclusion, we submit these cofactors as novel components of the ecdysone signaling pathway in order to further elucidate the dynamics of steroid specificity.

## Background

Steroid hormones regulate many developmental processes in higher organisms, including postembryonic development, metamorphosis, and reproduction [1-3]. Pulses of the steroid hormone 20-hydroxyecdysone (referred to from here on as ecdysone) direct the morphological transitions of *Drosophila *throughout its life cycle [4-12]. Titers of ecdysone increase before each postembryonic larval molt and it is required for triggering metamorphosis transitions [3]. One of the highest peaks of ecdysone triggers the transition from third larval instar to puparium formation at the onset of metamorphosis, which involves simultaneous down-regulation of cell death inhibitors and up-regulation of cell death activators in larval tissues while activating proliferation and differentiation cascades in imaginal tissues [2,3,13]. This increase in the ecdysone titer during puparium formation is transduced to the target gene level via an Ecdysone Receptor (EcR)/Ultraspiracle (USP) heterodimeric complex [14]. As established by the Ashburner model, this complex activates a cascade of transcription factors [3,14] called early genes [1,15-17] and concurrently represses a set of 'late genes'. These "early genes" coordinate the temporal and spatial activation of late genes, which then carry out the metamorphic process [14,17].

The EcR/USP heterodimer is a conserved protein complex that resembles several vertebrate nuclear receptor complexes. Thus, the discovery that the tissue specificity of vertebrate nuclear receptor transcription is mediated by coregulators led to identification of *Drosophila *nuclear receptor coregulators [18]. While many of the molecular mechanisms involving the ecdysone response are known, very little is known about the coregulators required for proper signal transduction and specificity. Because ecdysone controls varied functions in distinct tissue types, it is likely that specific cofactors interact with EcR in the various tissue types. We have sought to identify at least a subset of these unknown cofactors by utilizing a bioinformatically informed RNAi luciferase reporter screen.

Extensive evidence has shown that many steroid receptor cofactor proteins harbor an LXXLL motif, where L is leucine and × is any amino acid [19-21]. The LXXLL motif was first identified in proteins that are important in nuclear receptor (NR) regulation and specifically bind to the AF-2 region of nuclear receptor Ligand Binding Domains (LBDs) [19]. Subsequently, data continued to reveal that many steroid receptor coactivators that enhance transcriptional regulatory function of the NRs have interacting domains that contain highly conserved LXXLL motifs (LXD's)[22,23], and that these domains are both necessary and sufficient to mediate association of coactivators to ligand-bound receptors [24] by an alpha helical locking mechanism which causes the receptor to retain secure binding of the ligand. Conversely, steroid corepressors tend to contain extended LXD motifs, often some version of LXLXXL or LXIXXL, which impedes the binding of ligand molecules and thereby removing the transcriptional activation of the hormone signal [21]. The clinical and developmental necessity of these domains is further exhibited in familial disorders in humans, attributed to genetic variations which ablate these domains and impede steroid functions [25].

For the study reported here, we conducted an *in silico *pre-screen of the *Drosophila melanogaster *proteome to identify putative cofactor candidates that were then interrogated in an RNAi *in situ *luciferase reporter screen. Using prior knowledge of spatial structure amino acid context and abundance of LXD motifs in known coactivators and corepressors, we compiled a list of candidate EcR cofactors. Also, by including a computational screen of *Drosophila *proteome function and interaction databases [26-29], we filtered the candidate gene list for transcriptional function and/or known physical interaction with transcription factor complexes. Ultimately, this functional study included 95 putative EcR cofactors (Table 1) and we utilized four distinct cell lines to investigate tissue specificity of cofactor function. The cell lines included, two embryonic lines (Kc167 and S2) and two imaginal lines (L1 and D20), all of which were derived from unique tissue sources, in order to determine whether the specific cofactors are involved in either activation or repression of the ecdysone reporter gene in distinct tissue types.

**Table 1:** Cofactor candidate list and RNAi reporter screen results.

***Well ID***	**Symbol**	**Full Name**	**FBGN#**	**EcR Bound**	***Well ID***	**Symbol**	**Full Name**	**FBGN#**	**EcR Bound**
***A01***	Dfd*	Deformed	FBgn0000439	No	***E01***	CG5366^~^	CG5366	FBgn0027568	No

***A02***	babo^~ +^	Baboon	FBgn0011300	Yes	***E02***	MED16^~ ^^	Mediator complex subunit 16	FBgn0034707	No

***A03***	E2f2^~ +^	E2F transcription factor 2	FBgn0024371	Yes	***E03***	CG5899*	CG5899	FBgn0032157	Nd

***A04***	ptc^~^	Patched	FBgn0003892	No	***A06***	brm^~ +^	Brahma	FBgn0000212	Yes

***A05***	mys*^~^	myospheroid	FBgn0004657	No	***E05***	abo*	abnormal oocyte	FBgn0000018	No

***A06***	brm^~ +^	brahma	FBgn0000212	Yes	***E06***	HDAC6*	HDAC6	FBgn0026428	Yes

***A07***	**N/A**	**No RNAi**	**#N/A**	**Nd**	***E07***	Rfx^+ ^^	Rfx	FBgn0020379	No

***A08***	Hsp27^	Heat shock protein 27	FBgn0001226	No	***E08***	Taf2^+ ^^	TBP-associated factor 2	FBgn0011836	No

***A09***	Hr39	Hormone receptor-like in 39	FBgn0010229	No	***E09***	CG7154*	CG7154	FBgn0031947	No

***A10***	**Kr^~ +^**	**Kruppel**	**FBgn0001325**	**Nd**	***E10***	Acp36DE	Accessory gland peptide 36DE	FBgn0011559	Yes

***A11***	Myb* ^	Myb oncogene-like	FBgn0002914	No	***E11***	mus304* ^+^	mutagen-sensitive 304	FBgn0002901	No

***A12***	tst* ^~ + ^^	twister	FBgn0039117	Yes	***E12***	RecQ4*	RecQ4	FBgn0040290	Yes

***B01***	mia* ^	meiosis I arrest	FBgn0014342	No	***F01***	Orc5^+ ^^	Origin recognition complex subunit 5	FBgn0015271	No

***B02***	neb* ^	nebbish	FBgn0004374	Yes	***F02***	Sima	Similar	FBgn0015542	No

***B03***	eIF5B*	eIF5B	FBgn0026259	Yes	***F03***	MED17	Mediator complex subunit 17	FBgn0038578	No

***B04***	ida	imaginal discs arrested	FBgn0041147	Nd	***F04***	MED24	Mediator complex subunit 24	FBgn0035851	No

***B05***	CG11403*	CG11403	FBgn0026876	No	***F05***	Su(z)12*	Su(z)12	FBgn0020887	No

***B06***	CG11970* ^	CG11970	FBgn0027503	No	***F06***	Mi-2	Mi-2	FBgn0013591	No

***B07***	MED14*	Mediator complex subunit 14	FBgn0035145	No	***F07***	Tfb1	Tfb1	FBgn0033929	No

***B08***	tai* ^	**taiman**	**FBgn0041092**	**Yes**	***F08***	boss^+ ^^	bride of sevenless	FBgn0000206	No

***B09***	zfh2* ^	Zn finger homeodomain 2	FBgn0004607	Yes	***F09***	Jhe	Juvenile hormone esterase	FBgn0010052	No

***B10***	yemalpha	yemanuclein alpha	FBgn0005596	No	***F10***	CG8443^	CG8443	FBgn0034087	No

***B11***	Sara^	Smad anchor for receptor activation	FBgn0026369	No	***F11***	HLH106	Helix loop helix protein 106	FBgn0015234	No

***B12***	CG1582^~ ^^	CG1582	FBgn0030246	Yes	***F12***	Iswi	Imitation SWI	FBgn0011604	No

***C01***	fz3^	frizzled 3	FBgn0027343	No	***G01***	Asx*	Additional sex combs	FBgn0000142	No

***C02***	cnc^	cap-n-collar	FBgn0000338	nd	***G02***	eIF5* ^~ +^	eIF5	FBgn0030719	Yes

***C03***	InR^	Insulin-like receptor	FBgn0013984	Yes	***G03***	CG9323	CG9323	FBgn0032883	No

***C04***	mor^+ ^^	moira	FBgn0002783	Yes	***G04***	dom*	Domino	FBgn0020306	Nd

***C05***	sas^+ ^^	stranded at second	FBgn0002306	No	***G05***	hb* ^	Hunchback	FBgn0001180	No

***C06***	ush	u-shaped	FBgn0003963	Yes	***G06***	eIF3-S10*^	eIF3-S10	FBgn0037249	No

***C07***	CG2990	CG2990	FBgn0030170	No	***G07***	skd*	Skuld	FBgn0003415	Nd

***C08***	CG31212	CG31212	FBgn0086613	Nd	***G08***	Sin3A*	Sin3A	FBgn0022764	No

***C09***	kz*	kurz	FBgn0001330	No	***G09***	Tbp-1* ^+^	Tat-binding protein-1	FBgn0028684	Yes

***C10***	JhI-1* ^+^	Juvenile hormone-inducible protein 1	FBgn0028426	No	***G10***	Smr*	Smrter	FBgn0024308	No

***C11***	MED23^+ ^^	Mediator complex subunit 23	FBgn0034795	No	***G11***	MED1*	Mediator complex subunit 1	FBgn0037109	No

***C12***	kis* ^~^	kismet	FBgn0001309	No	***G12***	CSN5*	COP9 complex homolog subunit 5	FBgn0027053	No

***D01***	Chd1* ^	Chromodomain-helicase-DNA-binding protein	FBgn0016132	Yes	***H01***	Pros45	Pros45	FBgn0020369	No

***D02***	l(2)01424^	lethal (2) 01424	FBgn0010488	No	***H02***	Bx42^	Bx42	FBgn0004856	No

***D03***	trr	trithorax-related	FBgn0023518	No	***H03***	l(1)G0168^	lethal (1) G0168	FBgn0027287	Nd

***D04***	MED15^	Mediator complex subunit 15	FBgn0027592	No	***H04***	hyd^~ ^^	hyperplastic discs	FBgn0002431	Yes

***D05***	Hel89B	Helicase 89B	FBgn0022787	No	***H05***	Nipped-A	Nipped-A	FBgn0053554	Nd

***D06***	Bap60^	Brahma associated protein 60 kD	FBgn0025463	No	***H06***	spen^	split ends	FBgn0016977	Yes

***D07***	XNP	XNP	FBgn0039338	Yes	***H07***	RpL7A^~^	Ribosomal protein L7A	FBgn0014026	No

***D08***	Pk92B^+^	Protein kinase at 92B	FBgn0014006	No	***H08***	alien*^~^	Alien	FBgn0013746	No

***D09***	POSH^	Plenty of SH3s	FBgn0040294	No	***H09***	Hdac3^~^	Hdac3	FBgn0025825	No

***D10***	Rbf2^+^	Retinoblastoma-family protein 2	FBgn0038390	No	***H10***	**EcR^~ +^**	**Ecdysone receptor**	***FBgn0000546***	**Yes**

***D11***	CG5205	CG5205	FBgn0038344	No	***H11***	CG12129^	CG12129	FBgn0033475	No

***D12***	RfC3* ^~ ^^	RfC3	FBgn0032244	No	***H12***	Pc* ^~^	Polycomb	FBgn0003042	No

Listed in the order of orientation in the 96 well plate assay. Symbols in superscript next to gene symbols summarize the results from our RNAi screen for each cell type; * indicates the cofactor was a hit in D20, ~ indicates the cofactor was a hit in L1, + indicates the cofactor was a hit in Kc, ^indicates the cofactor was a hit in S2. In addition, columns indicating whether candidates are also binding targets for EcR based on published whole genome assays and the average expression level of each gene in the relevant cell types from data extracted from NCBI GEO (normalized expression units). Over 85% of the putative candidates were significant hits in our screen

## Results

### In silico pre-screen of Drosophila melanogaster proteome for putative cofactors; LXD motifs predict cofactor function

Our first goal, in experimental design, was to conduct a concise functional screen of deduced putative cofactors as opposed to a more expensive, data intensive and false positive stricken whole genome screen. Armed with compelling evidence of cofactor protein LXD domains from Yeast to Human and all in between, we have focused our cofactor search on proteins which harbor these extensively studied LXD motifs. Accordingly, we anticipate increasing the probability of finding actual cofactor hits in our reporter screen by capturing the putative cofactors which have the necessary structure to function as a steroid receptor cofactor. Recent directed mutagenesis studies have shown that the LXXLL (LXD) motif creates an AF-2 interaction domain necessary for coactivator function of specific steroid receptor complexes and is further characterized as having specific hydrophobicities and charged amino acid contexts [19,20,30-38]. Based upon this evidence, we utilized an extensive search for the LXD and extended LXD motifs throughout all proteins in the *Drosophila melanogaster *genome.

**Figure 1:**
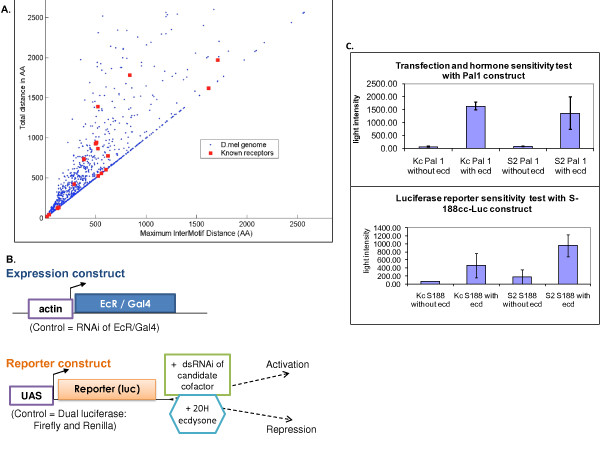
**Overview of *in silico *pre-screen and experimental design tests**. A. Scatterplot of LXD intermotif distances for the 2200+ genes identified in the proteome search. Red squares indicate the distributions for LXD motifs of known cofactors. Cofactors falling on the diagonal have only two motifs. B. RNAi screen schematic showing the construct of the expression and reporter plasmids. The screen is designed to detect endogenous cofactors that are necessary for the ecdysone response of the luciferase reporter gene. C. Assay transfection and reporter response control tests. Data shown indicates the system is ecdysone responsive as significantly higher levels of luciferase activity was detected cells treated with ecdysone.

We initially identified 4782 genes with at least one LXD according to the genome annotation version, Dmel r3.1 http://www.Flybase.org. Already having reduced the 15,000 gene genome by over 70%, we then utilized the functional protein models of known coactivators and corepressors to enhance our *in silico *screen for amino acid context. Our next level of filtering required the proteins to have more than one LXD within 200 amino acids [20,37,39-41] as the published steroid receptors all had at least two motifs (Figure 1A). In addition to these LXD motif requirements, we also incorporated the flanking regional properties of amino acid charge and hydrophobicity common to known nuclear receptor cofactors. By using these characteristics of known cofactor LXD's as pre-filtering parameters [37,38,40,42-47], we identified 130 genes which matched the requisite motif primary sequence, protein sub-structure and amino acid composition. This list of putative cofactors yielded a Gene Ontology enrichment for "positive regulation of transcription", with a Benjamini corrected p-value of 1.5^E-6 ^which further supported the impact of our proteome pre-screen.

In addition, similar to the LXD motif, extensive evidence has indicated that "extended LXD motifs" within corepressor proteins have an antagonistic effect on nuclear receptor transcriptional activity. Using similar algorithms developed for the LXD search, we conducted an *in silico *proteome screen for the extended LXD motif and found 563 genes with at least one extended LXD. Of these, only 24 had two or more motifs and we used each of these in our study. This list did not show a statistically significant enrichment of negative regulation after Benjamini correction; however, there was a clear enrichment of transcriptional regulation.

As another bioinformatics measure, we queried the NCBI GEO database to determine if our putative cofactors were all expressed in the cell lines of interest (Table 1). While not all candidates appeared to be expressed in each cell line, we did confirm that during the lifecycle, all genes were expressed at timepoints relevant to at least one pulse of ecdysone. Ultimately, our *in silico *screen did identify previously known EcR cofactors, such as Taiman and Kruppel, which we have included in our assay. This suggests our *in silico *search was successful in predetermining cofactors of the hormone receptor signal.

### The luciferase screen is ecdysone responsive

To ensure that our reporter screen would be effective in identifying EcR cofactors, test transfections were performed in Kc176 and S2 cell lines with Pal1sx-188ccLuc and S188cc-RLuc plasmids [48] to first ensure the transfections were successful and also that the *in vitro *reporter system is 20 hydroxyecdysone (ecdysone) responsive (Figure 1B and 1C). The expression constructs and the reporter construct architecture are depicted in Figure 1B. Cells transfected with the reporter construct alone displayed low levels of luciferase activity, indicating the transfection was successful (data not shown). However, since this plasmid is not under an EcRE promoter, there was no difference in luciferase activity in cells treated with ecdysone. Therefore, we employed an ecdysone sensitive test plasmid to measure the hormone sensitivity of the transfection/reporter system in each cell line (Figure 1C). The Pal1sx-188ccLuc construct harbors a 5X EcRE promoter and shows significant differences (over 10 fold) in luciferase activity between cells treated with or without ecdysone (Figure 1C - top). These results indicate that the cell line system is ecdysone responsive. We next tested the sensitivity of our actual assay plasmids. The co-transfection of the expression and reporter constructs also show ecdysone sensitivity and expected lower levels (due to lack of the 5X EcRE), but within 2-4 fold induction of reporter expression (Figure 1C- bottom).

### Distinct cofactor behavior among cell types

**Figure 2:**
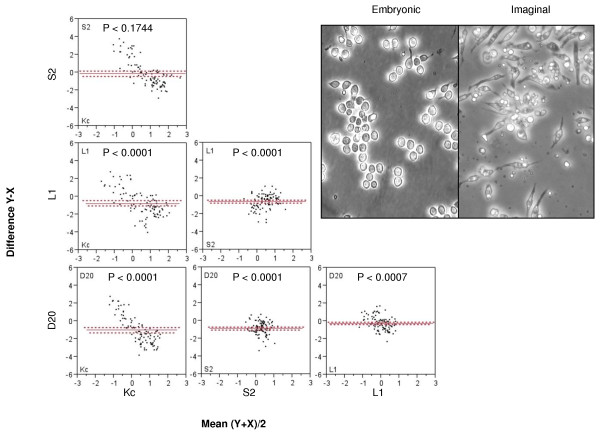
**Comparison of RNAi results for each cell type**. Panel A, Scatterplot matrix of paired differences of mean reporter expression values, for each RNAi target tested. Red solid line indicates median difference and dashed lines indicate confidence intervals. Each comparison indicates the cell lines have significantly different responses in overall reporter activity. The data show that each cell line's reporter responses across the screen were significantly different from all others across the assay, indicating the cell type specificity of cofactor action on the reporter gene. Inset, representative images of the embryonic (Kc/S2) and imaginal (D20/L1) cell lines displaying the general morphological differences between each tissue type.

We chose four cell lines for our study, representing two tissue types; imaginal discs and embryonic (Figure 2). Previous seminal ecdysone treatment studies indicate each cell type exhibits unique morphological responses to the hormone [49-51], correlating to the original tissue response which are also summarized in Flybase and the Drosophila Genome Resource Center references. These data suggest that different subsets of target genes are modified during hormone pulses within each cell type. In validation of this, a recently published study [52] shows distinct gene signatures in each of our cell lines, in response to the same ecdysone treatment. To accomplish this targeting of distinct genes by ecdysone in the organism, we hypothesize the different cell lines have unique cofactors which direct the receptor to targeted genes of specific functions and determine whether the target genes will be activated or repressed. Figure 2A depicts the pairwise statistical testing of each cell lines' mean differences of reporter gene expression in response to the RNAi knockdown of 95 putative cofactors (Table 1) following ecdysone treatment. The results show each cell line exhibited significantly unique reporter gene activity across the assay when compared to one another. This indicates there is considerable cell type specificity of cofactor actions on the reporter construct, which most likely correlates to the endogenous ecdysone target gene response.

**Figure 3:**
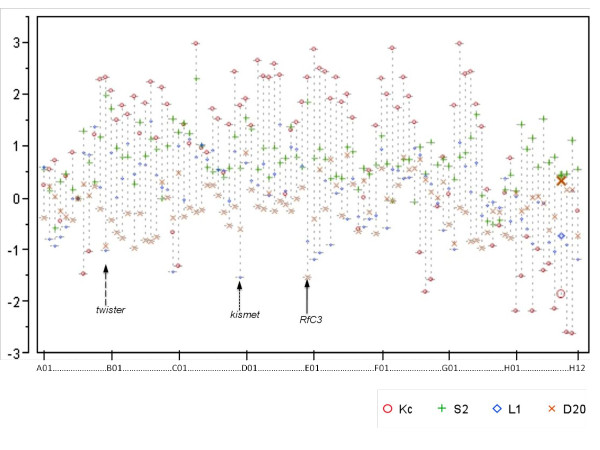
**Gene by gene overview of RNAi reporter gene expression**. The overlay scatterplot indicates the expression level changes in luciferase reporter values, relative to no RNAi, in each of the cell lines. Each marker represents a specified cell line and each candidate cofactor dsRNAi treatment is arranged along the × axis in the order listed within the assay plate by row (letter) and column (number), also indicated on Table 1. Arrows indicate cofactors which show opposing polarity of transcriptional regulation, specific to indicated tissue types. Long dashed arrow indicates, twister. Short dashed arrow indicates kismet. Solid arrow indicates RfC3.

Figure 3 displays a scatterplot of normalized reporter gene expression levels for each cofactor knockdown (in order of 96 well orientation, see Table 1) and color/marker coded by cell type. We see on a gene by gene basis that each of the cofactors tested have a varying range of reporter gene impact among the different cell types. Statistical testing indicated that each cell line had a significant number of unique reporter gene modulations, meaning within each cell type, each cofactor hit had a distinct reporter gene impact from the other cofactors (Figure 3) (i.e. no single cofactor had the same impact on reporter response across all cell types). Pearson correlation analyses indicate none of the cell line assays were significantly correlated; however, the two embryonic cell lines' (Kc and S2 = 0.1479) assays were relatively more similar when compared to the correlation scores against imaginal cell lines (-0.072) while L1 and D20 showed the same trend. This result is to be expected, as the lines are not derived from the same type of embryonic or imaginal disc origin tissues and we would expect the distinct differentiation mechanisms of each organ in response to ecdysone pulses requires unique cofactors for divergent organogenesis pathways.

### Bi-polar cofactors, tissue-type specificity of transcriptional coactivator vs corepressor regulation

**Figure 4:**
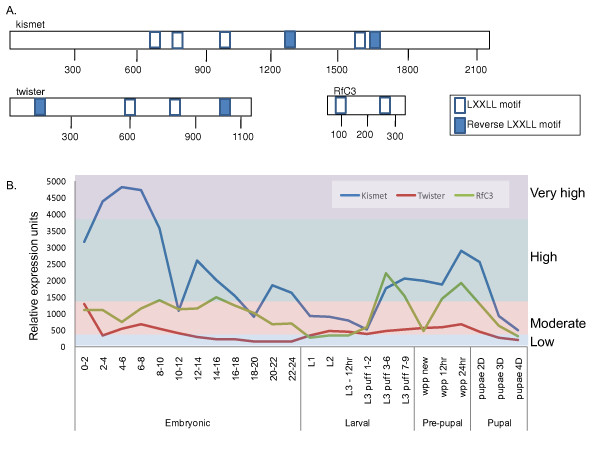
**Schematic of 3 common cofactor LXD motif and lifecycle expression**. A. Schematic of common cofactor LXD motif protein domains. B. Lifecycle expression patterns, data retrieved from "flyatlas" database. Colored panels indicate relative expression levels as low (blue), moderate (red), high (green) and very high (purple).

**Figure 5:**
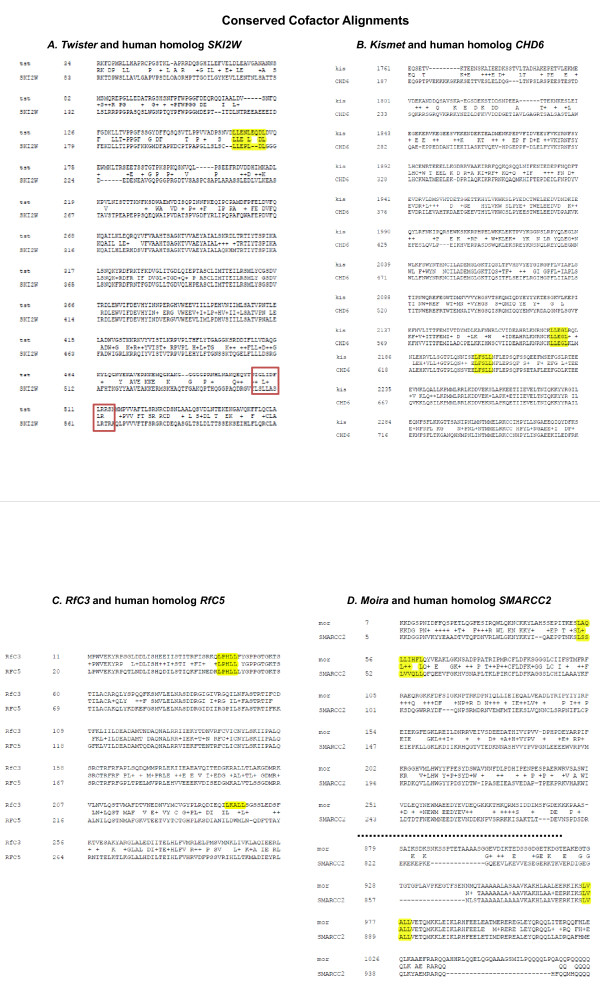
**Sequence alignments of three common EcR cofactors with their human homologs**. A. Subsection of Twister protein sequence contains a highly conserved extended LXD domain (highlighted orange) and a region of what may be an evolving LXD domain (red box). B. Kismet also contains two completely conserved LXD motifs (highlighted yellow). C. Moira and its human homolog SMARCC2 which is part of the well know steroid receptor cofactor complex SWI/SNF show presence of both LXD and extended LXD motifs. Both genes have been shown to be involved in both activation and repression of developmental target genes.

The nature of our experimental design allowed us to distinguish between coactivators and corepressors in our ecdysone inducible system. In fact, within some lines the removal of particular cofactors causes a higher level of hormone induced activation of the reporter, when compared to the no RNAi (well A10) while in other cell types it causes repression (Figure 3 and Table 2). We found that 9 cofactors function as both coactivators and corepressors in a cell type dependant manner. Cofactors which exhibit this bi-polar regulation effect harbor both the LXD and extended LXD motifs in their amino sequence (Figure 4A). Of these, three genes show the strongest tissue type correlation of polarity in reporter gene response where they have the same activation or repression conserved within embryonic, in contrast to having the opposite activity in imaginal cell types. These genes, Twister, Kismet and RfC3 show pulses of expression during both metamorphosis and embryogenesis (Figure 4B) and are each highly conserved proteins (Figure 5). This suggests that preferential usage of certain domains could be the mode of achieving differential target gene responses unique to certain tissue type.

**Table 2:** Coactivators vs Corepressors identified in embryonic vs imaginal cell lines.

**EcR Cofactors in Embryonic Lines**		**EcR Cofactors in Imaginal Lines**	
*Activators in Kc*	*Repressors in Kc*	*Activators in L1*	*Repressors in L1*
CG12129	Moira	kismet	Taiman
Ecdysone Receptor	CG9323	CG1582	XNP
Pros45	Mediator complex subunit 17	CG5366	Kruppel
Hyperplastic discs	CG5366	Polycomb	Insulin-like receptor
alien	trithorax-related	Mediator complex subunit 16	CG9323
Hdac 3	Brahma associated protein 60 kD	alien	Imaginal discs arrested
Juvenile hormone esterase	Mediator complex subunit 16	twister	Stranded at second
CG8443	hunchback	eIF5	frizzled3
Bx42	CG5899	E2F transcription factor 2	
Heat shock protein 27	Mediator complex subunit 23	brahma	
Splitends	domino	Hyperplastic discs	
Frizzled 3	XNP	Ribosomal protein L7A	
Ribosomal protein L7A	Mediator complex subunit 15		
Bride of sevenless	twister		
Hormone receptor-like in 39			
***Activators in S2***	***Repressors in S2***	***Activators in D20***	***Repressors in D20***
E2F transcription factor 2	moira	RfC3	Chormodomain-helicase
Tat-binding protein-1	twister	skuld	Rfx
Sin3A	RfC3	twister	Su(z)12
Smrter	meiosis1arrest	eIF5	Bride of sevenless
Tfb1	eIF3-S10	taiman	Brahma
Mediator complex subunit 17	Chromodomain-helicase	hunchback	Tfb1
Yema nuclein alpha	splitends	eIF3-S10	Origin recognition complex subunit 5
	CG1582	Mediator complex subunit 14	Mi-2
		COP9 complex subunit 5	
		eIF5B	
		Zn finger homeodomain 2	
		Polycomb	
		Mediator complex subunit 1	

The specific coactivators and corepressors identified in the RNAi reporter gene assays for each cell line as indicated.

### Cofactors elucidated from our screen are implicated in ecdysone signaling

Of the 94 cofactors tested 37 were implicated as cofactors in D20 cells, 18 were implicated as cofactors in L1 cells, 19 were implicated as cofactors in Kc cells, and 35 were implicated as cofactors in S2 cells (Table 2). In addition, there were several cofactors found to be shared among different cell types (Table 3).

**Table 3:** Common cofactors implicated in ecdysone signalling in multiple cell types.

**Cell Lines**	**Common Cofactors**	**Full Name**
*Imaginal Specific Cofactors* *(D20 and L1)*	alien kis Mys Pc	alien kismet myospheroid Polycomb

*Embryonic Specific Cofactors* *(Kc and S2)*	boss MED23 mor Orc5 Rfx sas Taf2	bride of sevenless Mediator complex subunit 23 moira Origin recognition complex subunit 5 Rfx stranded at second TBP-associated factor 2

*D20 and Kc*	Jhl-1 mus304 Tbp-1	Juvenile hormone-inducible protein 1 mutagen-sensitive 304 Tat-binding protein-1

*D20 and S2*	CG11970 Chd1 Elf3-S10 hb Mia Myb Neb Tai zfh2	CG11970 Chromodomain-helicase-DNA-binding protein eIF3-S10 hunchback meiosis I arrest Myb oncogene-like nebbish taiman Zn finger homeodomain 2

*Kc and L1*	Babo brm E2f2 EcR	baboon brahma E2F transcription factor 2 Ecdysone receptor

*L1 and S2*	CG1582 hyd MED16	CG1582 hyperplastic discs Mediator complex subunit 16

*D20, Kc, and L1*	Elf5	eIF5

*D20, L1, and S2*	RfC3	RfC3

*D20, Kc, L1, and S2*	Tst	twister

Seven of the cofactors tested appear to be embryonic specific while four appear to be imaginal cell specific.

Seven cofactors were common to both the embryonic lines, Kc and S2 (Table 3) and are therefore considered to be embryonic specific ecdysone cofactors. Several of these genes have highly conserved functions throughout the animal kingdom and a few are conserved even within plant and bacteria (Figure 5). The temporal and spatial expression patterns of our cofactors correlate with ecdysone pulses and EcR action. For example, *Rfx *is an RNA Pol II transcription factor that is expressed in the peripheral nervous system and brain during embryogenesis [53], is absent in the sensory organ precursors of imaginal discs, and then appears again after puparium formation [54]. *Mor *encodes for a component of the ATP-dependent chromatin remodeling BRM complex, and is thought to be essential for complex integrity [55,56]. *Mor *transcription is regulated by the DRE/DREF regulatory pathway, which is required for expression of genes involved in cell proliferation [56] and implicated in ecdysone regulation [57]. *MED23 *has RNA Pol II transcription mediator activity and is involved in transcription initiation from the RNA Pol II promoter. *MED23 *is a part of the Mediator complex and is required for differentiation-inducing factor and heat-shock factor mediated transcriptional activation [58] both of which are related to ecdysone regulation. *Orc5 *plays a crucial role in cellular proliferation through its involvement in DNA replication and chromosome condensation and organization during mitosis [59]. *Boss *encodes a G protein coupled receptor that is required for propter insulin signaling [60]. Taf2, a TATA-box binding protein-associated factor, is involved in transcription initiation from the RNA Pol II promoter and is essential for viability [61].

Four implicated cofactors were common to both imaginal lines, D20 and L1 (Table 3). Polycomb (*Pc*) is extensively known to form multi-protein chromatin complexes which maintain transcriptional repression of homeotic genes throughout development via chromatin remodeling and histone modification [62] and has recently been implicated in an ecdysone mediated regulation of neuronal remodeling [63]. Kismet (*Kis*) a member of the CHD subfamily of chromatin-remodeling factors is thought to stimulate early elongation by Pol II and counteract Pc group repression [64]. It has a temporal expression pattern which correlates with ecdysone activity (Figure 4) Myospheroid (*Mys*), which encodes the *β *integrin βPS, is involved in the behavioral responses to aversive and attractive odorants [65]. *Alien *has been shown to be a corepressor of nuclear hormone receptors EcR and TR, and is thought to mediate gene repression by recruitment of SIN3A [66]. Incidentally, in our study *Sin3A *was found to be an implicated cofactor in D20, the imaginal antennal disc line (Table 1).

*Twister (Tst) *is the only cofactor that was implicated in the EcR signaling pathway in all four cell lines. *Tst *has homology to both yeast and human RNA helicases and has protein motifs that are typical of the Superfamily II helicase family, and is thought to be involved in the 3'-5' mRNA turnover pathway in *Drosophila *[67]. *Tst *is expressed in two transcript variants which result in two protein products that vary in size and are differentially expressed throughout drosophila development [67]

*Babo*, a TGF-β/Activin type I receptor and an implicated cofactor in L1 and Kc lines, is known to mediate neuronal remodeling in *Drosophila *by upregulation of expression of the EcR isoform EcR-B1 [68].

## Discussion

### Evolution of LXD motifs may impact steroid receptor cofactor interaction and function

Previous studies have investigated the evolution of nuclear receptor cofactors and their amino acid sequence conservations [18,69-72]. Alignments of amino acid sequences with human homologs were done for the cofactors which were common among most of the cell types. Alignments revealed that these proteins' amino acid sequences were significantly conserved and their homologs also retained specific cofactor functions. Intriguingly, the Drosophila coactivator proteins investigated have LXD motif regions that are also present in human homologs indicating their potentially conserved function and also implying their importance in development (Figure 5). For instance two proteins which functioned as co-repressors in our screen (*Twister *and *Kismet*) harbor extended LXD motifs conserved in humans that would be necessary for receptor repression interaction, and their human homologs are also annotated to function as negative regulators of transcription [67,73]. In contrast, the Kismet human homolog is annotated (GO) to be an RNA helicase which may now implicate helicase activity for proper ecdysone regulation of target genes. In addition, as mentioned above, we were able to find co-existing LXD and extend LXD motifs in the 'bi-polar' cofactors that were also present in their human homologs. Indeed, at least one of these corepressors (*SKI2W*) plays a dual role as a positive regulator of transcription in human models and harbors both LXD an extended LXD motif (Figure 4), which is necessary for receptor activator interaction. In some cofactor LXD motifs, the human homologs appear to have only remnants of the LXD motifs and or development of new motifs. For instance, we found that several human coactivator homologs have a different structure of the LXD domains and/or an increase in the number of motifs. In at least one homolog comparison we find that the drosophila coactivator functions as a corepressor in humans and the LXD motif appears to have evolved into an extended LXD motif. This suggests that in conjunction with this transformed function, the human homolog protein structure has transformed as an extended motif in comparison to the drosophila protein which only has two short LXD motifs. This finding thus sets a stage to investigate the micro-evolution of LXD domains and correlating function of corepressor vs coactivator.

Due to the increased complexity of steroid signaling between flies and humans, in both variety and function, we would expect to see many sequence differences among homologs. The trend of conserved motifs and/or novel motifs that correlate with transformed transcriptional regulation polarity suggests there is potential to identify mechanisms of molecular evolution of steroid networks on a systematic level. For this study, we did not investigate whether each of the Drosophila coactivators, has a human homolog which could have transformed into corepressors through emergent extended LXD motifs (or vice versa). Such an investigation would be extremely intriguing and we will begin this interrogation presently. This type of in-depth analysis of transitioning motifs throughout the phylogenic tree can uncover the mode of functional transformation that leads to steroid signaling dynamics among different organisms and would be ground breaking in terms of evolutionary biodiversity and steroid signaling network dynamics.

### Conservation across all phyla indicates highly utilized and required cofactor function

**Figure 6:**
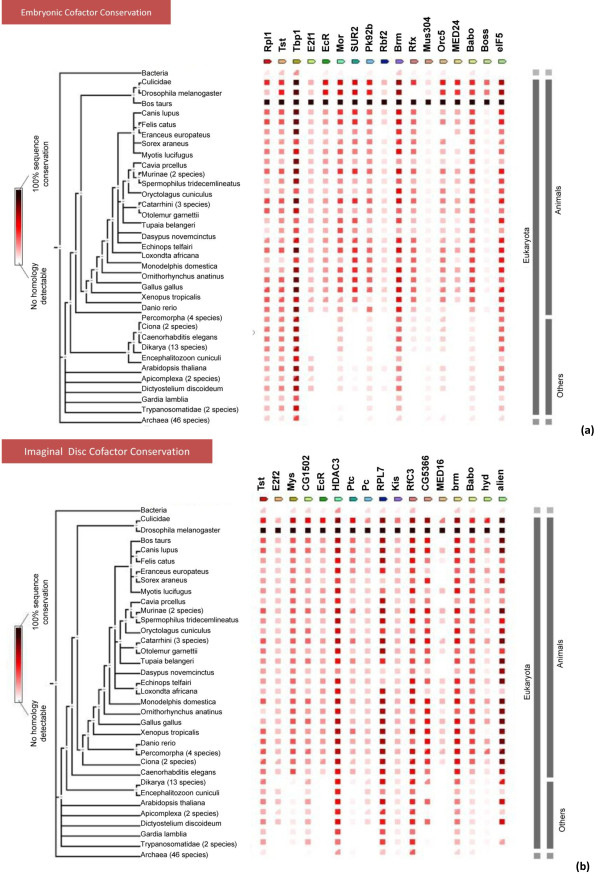
**Phylogenetic tree of top hits within the embryonic and imaginal disc cofactors**. Conservation score (by percent homology) is depicted as a heatmap, with darkest colors indicating 100% homology, see left inset for legend. A. Representative embryonic cofactor conservation tree. B. Representative imaginal disc cofactor conservation tree. Genes involved in general transcription and chromatin remodelling tend to have later/earlier conservation.

Several of the cofactor hits in our screens are highly conserved across all phyla (Figure 6) suggesting a highly conserved mechanism of tissue specific steroid target gene regulation. Indeed, steroid signaling is an integral part of development in all animals and even in plant morphogenesis, phytoecdysteroids play important roles [74]. With the presence of homologs in higher organisms and with such high scores of sequence conservation, we can assume that because these proteins have been conserved through so many layers of evolution that they are essential components of viability or development and these cofactor interactions, while still somewhat a mystery in terms of direct interactions and cell type specific impacts, are crucial to proper target gene regulation in response to steroidal cues. In fact, the most highly conserved proteins appear to be components in protein complexes which function in basic transcriptional machinery and chromatin remodeling, such as *brm, elf51 *and *tbp-1*. In contrast, some metamorphic specific genes are only moderately conserved throughout animals, such as *SUR2 and mys*, indicating these cofactors are probably exclusively functional in true steroid signaling regulation and transduction involved in morphogenic animal processes (Figure 6A and 6B respectively).

Further studies of homolog interaction with EcR homology receptors will uncover whether tissue specific cofactor-receptor interactions are maintained throughout different phyla. This will greatly impact and inform translational investigations which utilize model organism interrogation of hormone signals to elucidate human disease and treatment models.

### Networks of cofactor interactions may uncover the heterogeneity of hormone receptor complexes

**Figure 7:**
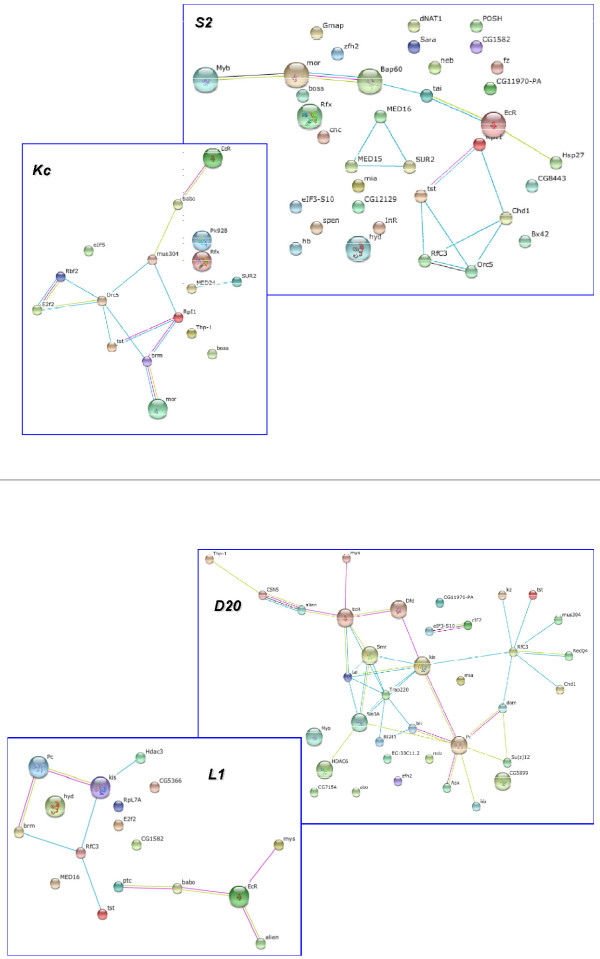
**Cofactor Interaction Networks**. Displayed are each of the genes which yielded a positive hit in our screen for the specified cell lines. The genes connected by indicated colored lines represent previously discovered interactions as curated in STRING databases as of 01/01/2011.

Previous studies have investigated the genome-wide interactions of proteins in Drosophila. We queried these and other protein interaction studies using the STRING interaction database [75-77]. We found that several of the cofactors we identified as significant to ecdysone regulation have previously been shown to interact with one another, either genetically or physically on the protein level. Figure 7 shows the previously identified interactions between the cofactors identified in our study. Clearly, there are several proteins which have not been identified as EcR related prior to our study; however, there are some intriguing associations which may uncover the nature of EcR complex heterogeneity among specific cell types. For instance, our data indicates that the strongest hit in our study, *Moira*, indirectly interacts with EcR through approximately 3-5 degrees of experimental separation (S2 and Kc). This involvement with ecdysone signaling through *brahma *complexes has been suggested prior to our cofactor screen [78] where ecdysone inducible genes were highly misregulated in the absence of *brahma *complex genes. Our study shows functional validation of this hormone signal interaction with the complex as several components of the *brahma *family have been implicated in ecdysone signaling. It appears, in our reporter system, its interaction requires components of the *brahma *complex.

Similarly, *mys *has been implicated in imaginal disc processes regulated by ecdysone and our study is the first to show valid transcriptional functional interaction. A phenotypic enhancement screen previously showed a low confidence single degree of separation between mys and EcR (GRID interaction database). In our screen, we find that mys is a significant hit in both imaginal disc cell lines, corresponding well with their specific tissue origin.

## Conclusions

### Steroid signaling cell type specificity... unraveling complexity

In our ecdysone inducible reporter system, we have clearly shown that the knockdown of over 80 putative candidate genes have a significant impact on reporter gene expression in a cell type specific manner, implying their cofactor activity with the endogenous EcR. We submit these cofactors as novel components of the ecdysone signaling pathway as coregulators to achieve spatial distinction of target gene regulation. For decades, it has been readily accepted that steroid signaling, while exposed to the entirety of an organism, is quite refined and directed in organ specific responses. Using a biologically informed *in silico *search, we have quickly identified and validated several of the cell type specific cofactors necessary for proper target gene regulation. While several of these genes have been implicated in ecdysone related process, this is the first time they are presented to have direct influence on ecdysone target gene regulation.

### Dynamics of tissue specific transcriptional polarity of cofactor interactions

While certain cofactors have exhibited a distinct coactivator or corepressor function, several have exhibited potential to play both roles in distinct cell types. To achieve the function of both corepressor and coactivator, we have confirmed that these proteins harbor both LXXLL domains (coactivator specific) and extended LXXLL domains (corepressor specific). These domains are also conserved in higher order homologs (Figure 5) along with conserved annotated function. This work has shed light upon molecular mechanisms of tissue specific protein interactions in the context of steroid hormone spatial specificity.

While we were unable to address this in our current study, we know that EcR has distinct isoforms with preferential expression in certain tissue types (i.e. EcR-B1 in larval tissues and EcR-A in imaginal tissues). We expect that the endogenous EcR is expressed during this assay and may interact with the reporter plasmid. Therefore, our data may indicate that the mode of action for specific cofactors is most likely not via the same direct interaction with the receptor and this perhaps could be due to different receptor isoforms having distinct AF-2 domains and therefore, exhibiting a distinct functional interaction potential with the cofactor. This work lays groundwork for identifying preferential protein domain interactions between specific cofactor LXD or extended LXD domains and the AF-2 domains of distinct steroid receptor isoforms. Such data would further unravel the mystery of tissue specific responses to hormone signals.

## Methods

### Drosophila cell culture and cell line derivation

The cell lines, S2, Kc157, D20c and L1 were utilized in this study. The S2 and Kc cell lines are both embryonic lines and two of the earliest established from Drosophila [79,80]. The S2 (S2-DRSC from DGRC) line exhibits properties and markers of macrophages while the Kc line (Kc167 from modEncode) exhibits markers of lymph gland cells and hemocytes. Lastly, the L1 and D20 cell lines are derived from distinct imaginal discs after the mid-third instar competency pulse [81-83]. The L1 (CME-L1 from DGRC) line was derived from leg imaginal discs while the D20c line (ML-Dm20c5 from the DGRC) was derived from an eye antennae imaginal disc. All cell lines were maintained at 25°C with no CO2 in 5 mL of media with the composition prescribed by the cell line source, Drosophila Genomic Resource Center (DGRC) https://dgrc.cgb.indiana.edu/cells/.

### Computational screen and candidate cofactor selection

A computational screen of the *Drosophila melanogaster *genome was conducted to determine putative cofactors. The program and pipelines utilized were customized by I. SanGil utilizing mixtures of Perl and Python scripting. Initially, more than 44,000 amino acid sequences were found to have the LXXLL motif, and a subset of these sequences was found to have between 2 and 22 of the LXXLL motifs. The majority of the known cofactors had between two and seven motifs that were within 1,000 amino acids of each other. In addition to these LXD motif requirements, we also incorporated the flanking regional properties of amino acid charge and hydrophobicity common to known nuclear receptor cofactors. By using these characteristics of known cofactor LXD's as filtering parameters we further delineated suitable cofactor candidates. Ultimately, these computational findings were used to create a list of the top 130 cofactor candidates to be studied further (Davis and San Gil, unpublished). The top ninety-six of these 130 cofactors were used in this study.

### dsRNA synthesis

dsRNA primer sequences were obtained from the Drosophila RNAi Screening Center at Harvard Medical School, which is now housed and distributed through the Drosophila Genomics Resource Center. These primers are the second generation design, and have previously been tested and confirmed to have no detectable off target effects. (Previous studies have established the efficiency of these sets to knock down transcription/translated levels of target genes, therefore it was not necessary to conduct such assays in our study. More information on OTE's can be found at the DGRC RNAi screening center website: http://www.flyrnai.org/DRSC-HOME.html). PCR was performed using 1 μM of the appropriate cofactor primer, 1.1 μg DNA and Platinum Taq. *In vitro *RNA transcription was performed using MEGAscript T7 kit (Ambion) according to the manufacturer's instructions. All product sizes were verified by running on a 1% agarose gel (data not shown). dsRNA was purified using Multiscreen PCR plates (Millipore) according to the manufacturer's instructions.

### Transfection and ecdysone treatment of cell lines

We utilized a dual luciferase reporter system (Firefly/Renilla), which enabled us to normalize reporter activity with expression of an unrelated constitutively expressed luciferase, which gives an indication of transfection efficiency. Cell lines were reverse transfected with either Pal15x-188ccLuc or S188cc-RLuc plasmids for sensitivity testing and the CMA-GBD-EcR-B1-N with UASx4-188ccLuc for the RNAi screen [84]. The Pal15x-188ccLuc plasmid contains a synthetic promoter enhancer region which harbors multiple adjacent copies of an EcRE for a known ecdysone target gene. This plasmid was mainly utilized to establish the ecdysone inducibility of our dual luciferase detection was reproducible in each cell line. The protein expression construct, CMA-GBD-Ecr-B1-N, is a fusion construct that contains an EcR ligand binding domain fused to a Gal4 DNA binding domain under the regulation of an actin gene promoter (Figure 1B). UASx4-188ccLuc contained the luciferase reporter gene driven by an inducible promoter via a Gal4 UAS sequence repeat. Cells were reverse transfected in a 96 well format with both constructs, pooled in equal amounts, and upon activation of the EcR protein by ecdysone treatment, the Gal4 DNA binding domain was expected to bind to the UAS and activate transcription of the luciferase gene (Figure 1). Pal1sx-188ccLuc contains the firefly luciferase reporter gene driven by an ecdysone-response element (EcRE). S-188cc-RLuc contains the enzymatically different Renilla luciferase and no detectable EcREs [84]. Transfections were performed using the FuGENE Transfection Reagent (Promega) with a 2:1 FuGENE Reagent: DNA ratio according to manufacturer's instructions. Three microliters of a [1 μg/ul] of ecdysone (20 Hydroxyecdysone - Sigma, H5142) was added 1 hour after transfection. 100 ng of the appropriate cofactor dsRNA was added 24 hours after ecdysone addition, therefore each screen took approximately two days.

### Luciferase assay and calculations

For each cell type, at least three replicates of the RNAi screen was completed using a reverse transfection protocol (above) in a 96 well plate format. Luciferase activity was detected 16-20 hours after addition of the dsRNA using Dual-Glo Luciferase kit (Promega) according to the manufacturer's instructions. For each dsRNAi gene, the reporter gene activity (k*_i_*) was calculated by creating an average ratio of Renilla ((Rlu) and Firefly luciferase (Flu), thereby normalizing for luciferase detection, across all replicates *(i-n)*. The luciferase reporter activity was then transformed into a change of reporter activity (Dk*_i_*) values by calculating the log_2 _ratio of the dsRNAi gene and the "no-RNAi" control wells' reporter activity. The log_2 _values of these ratios are shown in Figures 1 and 2, and were used for statistical analyses to identify significant changes in reporter activity due to RNAi of a candidate gene. Any cofactor that had a standard deviation greater than 2.0 relative to the no-RNAi control was selected as a significant cofactor. The no-RNAi controls were considered to be the normal ecdysone response value as these transfected cells were treated with ecdysone but not dsRNA. Fold differences of significant cofactors compared to removing EcR were then calculated by subtracting the EcR-RNAi value from the RNAi cofactor reporter value.

## Authors' contributions

MBD conceived experimental design and conducted RNAi assays and analyses. IS conducted all computations for *in silico *screening in collaboration with MBD. GB and RO conducted and assisted with all molecular wet bench work, including conducting replicates of assays and cell culture. LN assisted with writing the revised manuscript. All authors have read and accepted this manuscript for submission.
